# The status and the factors that influence patient safety in health care institutions in Africa: A systematic review

**DOI:** 10.1371/journal.pgph.0001085

**Published:** 2022-12-13

**Authors:** Kennedy Diema Konlan, Jinhee Shin

**Affiliations:** 1 Mo- Im Kim Nursing Research Institute, Yonsei University College of Nursing, Seoul, Korea; 2 Department of Public Health Nursing, School of Nursing and Midwifery, University of Health and Allied Sciences, Ho, Ghana; 3 College of Nursing, Woosuk University, Wanju-gun, Jeollabuk-do, Republic of Korea; The American University in Cairo, EGYPT

## Abstract

Poor patient safety practices may result in disability, injury, poor prognosis, or even death and are primarily associated with a common concern in Africa. This study synthesized the factors influencing the maintenance of patient safety in Africa’s healthcare institutions. There was an in-depth search in PubMed Central, CINAHL, Cochrane library, web of science, and Embase using the PICO framework. The search results were filtered for Africa and from 2011 to September 2021 to yield 9,656 titles after duplicates were removed using endnote software, and 211 titles were selected for full-text reading as 16 were selected based on predetermined criteria. The quality appraisal was done using the Mixed Methods Appraisal Tool. A matrix was developed, discussed, accepted, and used as a guide for the data extraction. A convergent synthesis design was adopted for data analysis as the data was transformed into qualitative descriptive statements. Patient safety ratings ranged from 12.4% to 44.8% as being good. Patient safety was identified as an essential structure to improve patient outcomes. The factors associated with patient safety were level of education, professional category, hours worked per week, participation in a patient safety program, reporting of adverse events, openness in communication, organizational learning, teamwork, physical space environment, exchange of feedback about error, and support by hospital management. Poor patient safety environment could lead to the staff being prosecuted or imprisoned, lack of respect and confidence by colleagues, embarrassment, loss of confidence and trust in the health team by patients, documentation errors, drug errors, blood transfusion-related incidences, development of bedsores, and disability. These strategies by health institutions to promote patient safety must focus on reducing punitive culture, creating a culture of open communication, and encouraging incidence reporting and investigations to ensure continuous learning among all health care professionals.

## Introduction

The advancement in medical practice is associated with many risks that can be detrimental to the patient and society. This may result in disability, injury, or even death and is associated mainly with unsafe care practices [1]. Patient safety is a new health discipline aimed at reducing harm in patient care and service delivery. Patient safety is defined by the World Health Organization (WHO) as the prevention of medical errors and side effects to patients or reducing harm to patients [2–4]. The harm that results from poor patient safety practices has led to the broader recognition of the importance of patient safety tenants in care delivery, its incorporation into the strategic plans of healthcare organizations, and growing research interest in minimizing harm and promoting safe practice [1, 5]. Patient safety actions aim to ensure that patients receive care congruent to standard practices and likely lead, if any, to minimal harm [1]. Issues related to patient safety were first raised in a classical book titled “To Err is Human: Building a Safer Health System,” which warned of the dangers of unsafe practice and emphasized safety as a key fundamental tenet of practice [6]. The harm resulting from unsafe practices is pervasive in the entire world. Previous studies show that 16.6% of all hospitalized patients in Australia and 3.7% of American patients were affected by adverse side effects and that 1 in 20 prescriptions in primary care are error-prone [7].

The incidence and prevalence of patient safety interventions in healthcare institutions appear to be on the ascendancy as studies show that about 10% of patients are usually harmed [8, 9]. Many factors (latent and active, system and individual, etc.) lead to patient safety incidents [8]. It was reported that about 14% of patients affected by poor safety practices sustained a permanent disability, 16% moderate disability, 30% minimal disability, and 8% unspecified disability [9]. In a systematic review, most contributory factors that were identified to influence patient safety practices irrespective of hospital setting or methodology were active failures or individual factors [10, 11]. Therefore, health care institutions must develop favorable patient safety as a culture to be imbibed and practiced by all professionals and patients. Patient safety culture is a deliberate way of life that ensures the safety of the patient and the care providers, including any person found within the care environment [12, 13].

The impact of poor patient safety practices is noted to be worst in Africa and the Mediterranean areas, where it was identified as an outcome of harm [1, 9]. Limited studies specifically discuss the factors associated with patient safety in developing countries [14]. Due to this limited literature on patient safety in developing countries like Africa, little is known about the influence of unsafe care and the culturally appropriate measures to curtail these actions. The studies that target patient safety mostly aim to estimate the incidence of harmful practices and are mainly cross-sectional. In line with this, the exact magnitude of patient safety issues in developing and transitional countries is generally unknown, even though patient harm-related issues can be classified as a global health problem [9]. It is necessary to confirm the status of patient safety culture research conducted in Africa and the factors of patient safety more clearly. Therefore, we conducted a systematic review to comprehensively investigate Africa’s patient safety culture and patient safety factors.

## Materials and methods

### Search strategy

Using predetermined keywords, five electronic databases were searched (PubMed Central, Cumulative Index for Allied Health Literature—CINAHL, Cochrane Library, Web of Science, and Embase). The keywords were developed guided by the Population, Intervention, Comparison, and Outcome (PICO) framework. The search was done using the appropriate Boolean operators, wildcard, and truncation where it was appropriate. Using the PICO framework, the populations were patients OR clients OR care recipients. The intervention was health safety OR safety culture OR hospital safety OR healthcare safety OR safety climate OR safet* environment OR injury prevention OR patient safety. The comparison was hospital OR nursing OR healthcare worker OR teaching hospital OR primary healthcare, OR clinic OR government hospital OR private hospital and the outcome patient treatment OR treatment outcome* OR health outcome OR health results OR care impact OR organizational culture.

### Search results

**Fig 1:**
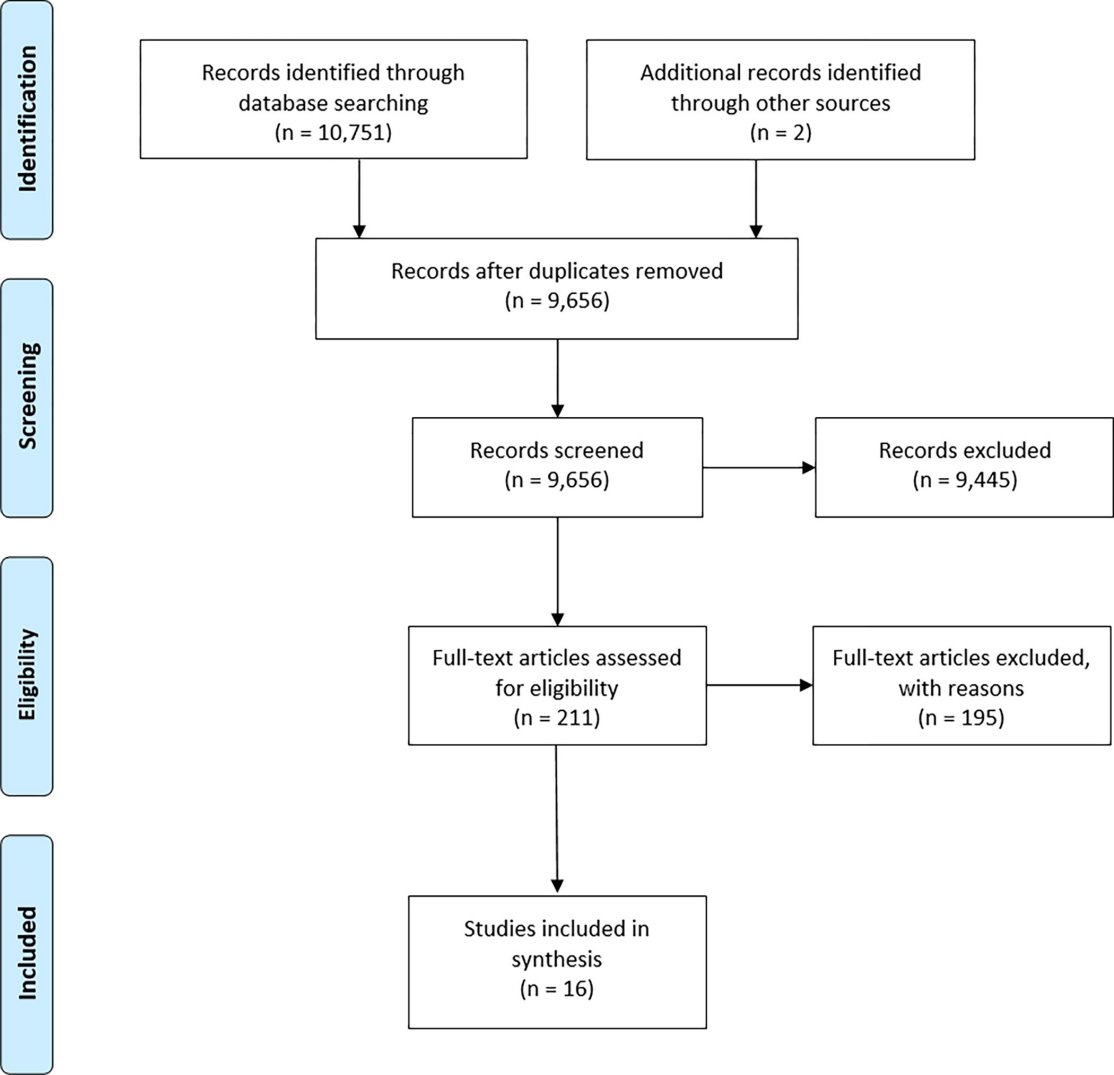
PRISMA flow diagram, article selection process.

There were in-depth searches in five electronic databases using the PICO framework and filtered for Africa in the last ten years (September 2021). The results produced 10,751 titles that were from PubMed Central (344), Cumulative Index for Nursing and Allied Health Literature—CINAHL (10), Cochrane library (734), web of science (1,903), and Embase (8,091). All the identified titles were transferred to endnote 20, and 1,097 duplicates were identified and removed; 9,654 titles remained for screening as shown in Fig 1. A priori inclusion criteria included African-based papers, studies assessing patient safety, those focusing on only health facility-based patient safety issues, and articles written in English. In contrast, the exclusion criteria were mainly non-facility-based patient safety studies. The study results were reported in line with the PRISMA checklist (S1 Checklist).

### Quality appraisal

The Mixed Method Appraisal Tool (MMAT) is a quality assessment tool that evaluates research’s methodological quality of qualitative, quantitative, and mixed-method research. Two researchers independently assessed the quality of each study using MMAT version 2018 [15]. The two researchers compared the evaluated data, and similarities were confirmed. Any discrepancies found between the two researchers were discussed until a consensus was reached.

The MMAT firstly has two questions that assess the clarity of the research question and whether the data addressed the specific research question handled by the data. All the selected studies met these screening criteria and then were designated to the area of the MMAT for quality appraisal. It was shown that thirteen studies were evaluated under the descriptive quantitative studies section. The section that deals with descriptive cross-sectional quantitative studies have sections that assess the relevance of the sampling to the research question, the representative of the sample to the target population, appropriateness of the measurements, the risk of nonresponse is low, and whether the statistical method adopted responded appropriately to the research question. All the studies identified in this category had all the responses being affirmative to each type except three [14, 16–26]. The three studies failed to meet the criteria requiring the study sample to represent the population [17, 19, 26]. It was noted that two studies [27, 28] evaluated qualitative studies. They were all found to be affirmative for the five categories of evaluation that included the qualitative approach answer the research question, adequacy of the qualitative data collection methods to the research question, if the findings are adequately derived from the findings, the interpretation of results is sufficiently substantiated by the data and if there was coherence in the qualitative sources of data, collection, analysis, and interpretation of findings. Only one study was evaluated as a randomized control trial [29]. It was affirmative for the five criteria questions that assessed if randomization was appropriately performed, if the groups are comparable at baseline, complete outcome data, blinding of assessors for intervention provided, and if the participants adhered to the assigned intervention.

### Data extraction

To ensure comprehensiveness and reproducibility of the data extraction process, a matrix was first developed, discussed, and accepted by both authors to use as a template for the data extraction. The two authors individually and independently extracted data from each study. The extracted data were then compared and streamlined. If there was a discrepancy in the process: a third person was invited to read the said article and arbitrated. The disagreement was resolved through consensus. The purpose of the data comparison process allowed for the comprehensiveness of the data extracted and allowed for clarity and reduction in ambiguity. The main parameters that constitute the matrix were the author and year of publication, objective, design, outcome variable, population, sample and sampling, measurement tool, factors affecting safety, and the study’s key findings.

### Data analysis

For the analysis of the data, a convergent synthesis design was adopted. Prior to this, the data were transformed into qualitative descriptive statements [30]. The thematic approach to qualitative data analysis was then adopted to conduct the convergent synthesis. There was a line-by-line coding of the various transformed qualitative descriptive statements independently as free codes by the two authors. Related and similar codes were then collated into subthemes. The sub-themes further coalesced into the main themes that emerged from the study. The main themes and sub-themes that emerged from the analysis formed the framework for presenting the findings. The first theme was the concept of patient safety with -sub-themes to include a rating of patient safety in the institution, attributes and dimensions of patient safety, sources of information on patient safety culture, and awareness/knowledge of patient safety culture. The second and third themes were the prevalence of patient safety incidence and repercussions associated with patient safety culture, which has sub-themes as negative repercussions of poor patient safety and positive consequences of good patient safety culture, respectively. The other main themes were factors associated with patient safety, challenges related to patient safety culture in the healthcare facility, and factors that promote patient safety practice in the healthcare facility.

## Results

### Study characteristics

The study approaches were qualitative [19, 28], quantitative [14, 16, 17, 20–26, 31], and intervention studies [29]. The specific study designs were cross-sectional [14, 16, 17, 19–24, 26–28, 31], descriptive correlational [25], randomized control trial [29] as shown in Table 1. The populations used were nurses [14, 20, 25, 27], physicians [14, 24, 27, 31], surgical team members [24, 28], paramedical staff and community pharmacists [14, 26, 31], clinical service staff or health worker [14, 16, 21–23, 27], administrative staff [27], physiotherapist students [17], and managers [27]. Others used whole facilities as sample units [19, 29] and community-based pharmacies [26], and volunteers [14], as indicated in Table 1.

**Table 1:** Distribution of study characteristics and consequences of patient safety.

Ref	Goal	Design	Country	Outcome	Population and sample	Tool	Prevalence and consequence
Aveling et al., 2015 [27]	Identify and explain the major obstacles to the safety of patients in care.	Cross-sectional	Two countries in East Africa	Environment, equipment, supplies, staffing, and teamwork	Hospital staff in two government hospitals Size (57)	Self-developed Semi-structured interviews	**Negative:** Poor teamwork and conflicts among various professionals. **Positive:** Lead to reduced patient harm and emphasize the quality of care.
Nwosu et al., 2019 [24]	Knowledge, attitude, and associated factors towards patient safety	Cross-sectional	Nigeria Enugu	Knowledge, attitude, and factors associated with patient safety	Nurses Size (386)	Self-developed pretested questionnaire	Important safety parameters were perceived hospital-acquired infection and abuse of transfusion. **Negative:** The risk of having the wrong surgery performed on a patient.
Labat & Sharma, 2016 [28]	Identified potential barriers to patient safety interventions	Cross-sectional	Eastern DRC	Barriers to patient safety interventions	Surgical team members Size (16)	Self-developed interview guide	**Negative:** Dire consequences on the health system, human resources and hospital management, and poor healthcare access. **Positive:** Lead to the presence of professionals and increased health worker resilience.
Swart et al., 2015 [25]	Educational background of nurses and their perceptions of quality of care and patient safety	Descriptive correlational	Gauteng Province in South Africa	Nurses’ perceptions of patient safety and quality of care	Nurses Size (149)	Self-developed questionnaire	Enrolled nurses rated all patient safety as very good (51.0%) and acceptable (51.0%). **Negative:** Medication errors, falls, and pressure sores.
Kumbi et al., 2020 [21]	Patient safety culture and associated factors among health care providers	Cross-sectional	Ethiopia in Bale Zone hospital	Patient safety culture, patient safety grade, and the number of events reporting	Healthcare workers Size (518)	Hospital Survey on Patient Safety Culture (HSOPSC)	The overall level of patient safety culture (44.0%). **Negative**: Adverse events
Moda et al., 2021 [22]	Occupational safety climate among healthcare workers in low- and middle-income countries.	Cross-sectional	Nigeria	Safety climate perception	Healthcare workers Size (433)	Nordic safety climate questionnaire (NOSACQ-50)	**Positive**: There is the active promotion of a positive safety climate in healthcare sectors. Likely to engage in positive safety behavior.
Gqaleni & Bhengu, 2020 [20]	Patient safety incident reporting system.	Cross-sectional	KwaZulu-Natal, South Africa	Types and frequencies of patient safety incidents	Registered nurse Size (224)	Patient Safety Incidents (PSIs)	Patient safety incidence was insignificant (18.0%) minor (35.0%), moderate (25.0%), major (12.0%), and catastrophic (10.0%).
Cheikh et al., 2016 [31]	Level of ‘patients’ safety culture among healthcare professionals.	Cross-sectional	Tunisia, Farhat Hached Sousse	Patient safety culture	116 licensed physicians and 203 paramedical Size (319)	HSOPSC	Event reporting (68.8%) and management support for patient safety (32.7%) **Negative:** Blood-related incidents (5.0%), medication-related events (7.0%), Ventilator-Associated Pneumonia (30.0%), multi-drug resistance (80.0%), and development of bedsores (78.0%).
Alhassan et al., 2015 [29]	Health service quality improves patient safety and risk reduction efforts by staff.	Randomized control trial	Ghana Western region	improvement in patient safety and risk reduction	64 primary healthcare facilities Size (16 offices)	Five primary risk areas in an assessment tool kit	**Positive:** Significant increases were recorded in nurses, laboratory technologists, pharmacists, and support staff. The average number of wards and laboratories per clinic increased significantly at follow-up.
Akologo et al., 2019 [16]	Healthcare providers’ perceptions of patient safety culture	Cross-sectional	Ghana Upper East	Perception of patient safety culture	Clinical staff Size (406)	HSOPSC	The average positive response for the 12 patient safety culture dimensions was 58.1%.
Yismaw et al., 2020 [26]	Patient safety culture of community pharmacists.	Cross-sectional	Ethiopia, Northern	Perception of patient safety	Staff of community pharmacies Size (120)	Pharmacy survey on patient safety culture (PSOPSC)	The overall percentage of positive responses on 11 dimensions ranges from 45%-90.2%, with an average percent positive response of 68.1%. **Negative**: Lead to poor communication work, pressure, and poor documentation of adverse events.
Mohammed et al., 2021 [23]	Patient safety culture and associated factors among health care professionals	Quantitative study	Ethiopia, northeast	Patient safety culture and associated factors	Health care professionals Size (422)	HSOPSC	The participants (44.8%) indicated a good patient safety culture.
Atakora et al., 2021 [17]	Level of knowledge, perception, and attitude of patient safety.	Cross-sectional	Ghana	Knowledge and perception of patient safety	Clinical year physiotherapy students Size (80)	WHO medical school curriculum guide for patient safety questionnaire	Most of the respondents (97.5%) had a moderate knowledge of patient safety. The majority (70.0%) of the respondents showed a moderate level (15–27) of knowledge about the error and patient safety, 10 (12.5%) indicated a low (7–14), and the remaining 14 (17.5%) showed high (28–35) levels.
Mayeng & Wolvaardt, 2015 [14]	Analyzed the factors that influence patient safety culture.	Cross-sectional	South Africa	Factors that influence patient safety culture	Health care professionals and volunteers Size (200)	The standard Manchester patient safety framework questionnaire	Patient safety was acceptable (42.4%), very good (28.5%), and excellent (14.6%).
Ente et al., 2010 [19]	Experience, awareness of medical error, and willingness to participate in patient safety initiatives.	Qualitative survey	Nigeria and Uganda	Awareness and experience of patient safety	60 healthcare professionals in 2 private and two public hospitals Size (80)	Questionnaire from the patient safety and healthcare quality literature	The frequency of occurrence of patient safety errors was 30.0%. **Negative:** Staff depression, guilt, and remorsefulness. Staff prosecution and imprisonment and loss of license. Lack of respect and confidence and trust, and embarrassment.
Gizaw et al., 2018 [18]	Perception of patient safety practice and associated factors	Cross-sectional	Jimma	Perception of patient safety	Healthcare providers in 5 hospitals Size (306)	Perception of patient safety practice	The overall perception of patient safety was (36.8%).

### The concept of patient safety

It was identified that two broad conceptualizations are associated with patient safety practices in health care facilities, including the need to avoid harm to patients and emphasize the quality of care rendered to patients and families [27]. These two conceptualizations emphasize health workers’ responsibility to the patient and the care process [27].

### Rating of patient safety in the institutions

It was identified that in Ethiopia, participants gave 12.4% and 29.3% rated patient safety grades as excellent and poor, respectively [21], and 44.8% showed good patient safety culture [23]. In South Africa, registered nurses perceived the quality of patient safety care to be adequate and desirable [25]. In Ghana, the safety culture engagement identified as an essential tool was described as the structured use of existing community groups to assess healthcare quality in health facilities [29]. Some graded patients’ safety within their units as acceptable (42.4%), very good (28.5%), excellent (14.6%), while 11.8% poor and 2.8% showed it was failing [14]. About 35.0% of the respondents perceived patient safety in their units as acceptable, while 13.8% and 1.0% perceived patient safety as poor and failing, respectively [16]. In Jimma hospital in Ethiopia, the overall perception of patient safety was (36.8%) [18]. In Ghana, the general perception of patient safety, 7.0% (n = 27) of the respondents perceived patient safety in their units as excellent, and 43.8% of the respondents perceived patient safety in their units as very good [16].

### Attributes/Dimensions of patient safety

The attributes that were identified to be associated with patient safety were hours worked per week, participation in a patient safety program, reporting of adverse events, communication openness, teamwork within the hospital, organizational learning, and exchange of feedback about the error [21]. In a survey of surgeons, hospital-acquired infection (64.0%) was considered an important issue related to patient safety. In comparison, others (34.0%) identified the overuse of blood transfusion services as an important issue in patient safety [24]. In Ethiopia, community pharmacists showed a high positive response rate demonstrated in the domains of teamwork (90.2%) followed by physical space and environment (83.1%) [26]. In Jimma hospital in Ethiopia, teamwork within the unit is the only area with above 75.0% positive response score (79.4%). Other areas with a composite percentage of positive response below 50% were frequency of event report (28.3%), hospital management support for patient safety (34.8%), hospitals handoffs and transition (41.4%), non-punitive response to error (44.8%), teamwork across the unit (47.4%) and communication openness (48.8%) [18]. There were five domains where the results were significant: overall commitment to quality dimension (*p* = 0.031); investigating patient safety incidents (*p* = 0.028); organizational learning following a patient safety incident (*p* < 0.001); communication about safety issues (*p* = 0.046); and team working around safety issues (*p* = 0.019) [14]. In Ghana’s upper east region, two dimensions of patient safety culture recorded the highest scores and included teamwork within units (81.5%) and organizational learning (73.1%) [16]. Doctors were consistently negative about all nine patient safety dimensions, while nurses were lukewarm in their responses on eight of the dimensions [14]. The results indicated that the community service staff had poor opinions on almost all nine dimensions. The communication about safety issues scored particularly poorly at 74.2% (*p* = 0.001) [14].

### Source of information on safety culture

Health care providers showed that the primary source of information on safety culture was experienced (50.5%), medical school (37.7%), the general culture (25.8%), and media (22.0%) [31].

### Awareness/Knowledge of patient safety culture

At the University of Ghana, most respondents (97.5%) had a moderate knowledge of patient safety [17]. In two hospitals in Nigeria and Uganda, frontline staff have good knowledge and understanding of medical errors [19]. Perception of patient safety practices increases by 0.168 as teamwork across the unit score increases by a unit (*p* = 0.023, 95% Confidence Interval (CI) = 0.02–0.31), by 0.113 (*p* = 0.026, 95% CI = 0.01–0.21) [18]. In two hospitals in Nigeria and Uganda, the staff is aware that errors could cause suffering to the patient and even lead to death [19]. The results also showed that medical error could lead to the staff being prosecuted or imprisoned, lack of respect and confidence by colleagues, embarrassment, loss of confidence, and trust in the staff by patients, the management, and the community [19]. In Ghana, there was no significant association between the level of study and knowledge of clinical year physiotherapy students on patient safety (*p* = 0.712) [17]. Participants pointed out that knowledge does not necessarily lead to good surgical practice for reasons ranging from lack of will, expressed as laziness and lack of dynamism, to lack of means [28]. The majority (72.5%) of respondents had a high level of knowledge regarding safety in the workplace, 22 (27.5%) respondents had a moderate level, and 78 (97.5%) respondents had a moderate level of overall knowledge of patient safety [17]. There was a strong correlation between the surgeon’s years of experience and the knowledge and utilization of institutional protocols to ensure patient safety in the health care institution among surgeons [24]. Consultants/specialists were about four times (Adjusted odds ratios (AOR) = 3.5, 95% CI = 1.92–6.64), and resident doctors were almost three times (AOR = 2.5, 95% CI = 1.24–4.87) more likely to have a good perception of patient safety issues than interns/ house officers [24]. In Ghana, respondents (60.0%) indicated a high knowledge of the safety of the healthcare system, while 40.0% showed a moderate level [17].

### Prevalence of patient safety incidence

In South Africa, the level of significance of patient safety was reported as 18.0% insignificant, 35.0% minor, 25.0% moderate, 12.0% major, and 10.0% catastrophic [20], as the overall level of patient safety culture was reported as 44.0% in Ethiopia [21]. In a multi-country study of patient safety in healthcare institutions following an outbreak of COVID-19 pandemic, worker safety commitment within the healthcare facilities was statistically significantly higher than management safety priority, commitment, and competence [22]. The classification of Patient Safety Incidence (PSI) in South Africa based on facilities showed that PSIs were classified into six categories: hospital-related incidents (42.0%); patient care-related incidents (30.0%); death (12.0%); medication-related incidents (7.0%); blood product-related incidents (5.0%) and Procedure-related incidents (4.0%) [20]. In Nigeria and Uganda, 30.0% of the participants said errors frequently occur, while only 3.3% were unsure how often errors occur in their hospitals [19]. The global percentage of positive responses was highest for frequency of event reporting (68.8%), supervisor/manager expectations and actions promoting safety (68.1%), and lowest for hospital management support for patient safety (32.7%) [31]. Good patient safety culture was positively associated with primary hospitals (AOR = 2.56, 95% CI = 1.56–4.21) [23]. In terms of how often these errors occur, 18 (30.0%) of them frequently (23.3%) occasionally, and the same number rarely said [19].

### Repercussion associated with patient safety culture

The repercussions of patient safety culture were either positive or negative. The positive where those things that will require a good patient safety culture are adhered to, and the negative results are when there is poor patient safety culture.

### Negative repercussions of poor patient safety

One of the adverse effects of poor patient safety practices was the risk of having the wrong surgery performed on a patient [24]. Blood-related incidents (5.0%) and medication-related events (7.0%) were more minor or insignificant, as most of the time, the correction measures were successful [20]. It was also observed that Ventilator-Associated Pneumonia (VAP) was the primary cause of death in neonatal Coronary Care Units (CCUs) (30.0%). Multi-drug resistance (80.0%) and the development of bedsores (78.0%) were the most reported PSIs in multidisciplinary CCUs [20]. Among community pharmacists in Ethiopia, there is no documentation in 59.0% of cases when a mistake that could have harmed the patient is corrected before the medication leaves the pharmacy [26].

### Positive repercussions of good patient safety culture

One result of patient safety within the health care institutions was the presence of professionals committed to their roles in service delivery [28]. In Ghana, safety culture engagement showed that interventions significantly enhanced leadership processes and accountability [29]. The nurses scored only substantially positive organizational learning following a patient safety incident (62.9%). Doctors scored the highest on staff education and training within their group about safety issues, the least poorly (58.3%) [14]. In Ghana, interventions to improve patient safety in health care facilities showed increasing patient safety and reducing risk significantly enhanced in intervention facilities primarily in the areas of leadership/accountability (Coef. = 10.4, *p* < 0.050) and staff competencies (Coef. = 7.1, *p* < 0.050) [29].

### Factors that are associated with patient safety culture

It was also noted that in the Democratic Republic of Congo, economic issues remain a significant challenge to patient safety from the health care system, human resources, hospital management, and patient access to health care services [28]. It was also noted that the surgical team members were more interested in a paternalistic organization structure and blame culture accompanied by inefficient support and low remunerations [28]. In South Africa, there was a significant statistical difference between nurses’ level of education (registered nurses versus enrolled nurses) and their reported knowledge of patient safety practices [25]. Factors associated with patient safety in a survey of health care providers were physician category of staff position; hours worked per week, primary work area (surgery and pharmacy), participation in the patient safety program, and adverse event reported showed association [21]. A significant effect of the management role was found regarding management safety priority, commitment, and competence [22].

In South Africa, several quality dimensions were statistically significant for the employment profile: overall commitment to quality (*p* = 0.001); investigating patient incidents (*p* = 0.031); organizational learning following incidents (*p* < 0.001); communication around safety issues (*p* = 0.001); and team working around safety issues (*p* = 0.005) [14]. The management safety justice dimension was found to have a high, statistically significant correlation to management safety empowerment (r = 0.68, *p* < 0.001) among the participants [22]. Doctors showed that the dimensions that influence patient safety culture were the dimensions that received lower positive response rates were hospital management support for patient safety (13.9%) and teamwork within units (45.4%). In comparison, those with the highest positive response included supervisor expectations and actions promoting safety (82.3%) and frequency of event reporting (84.0%) [31]. On the other hand, good patient safety culture was negatively associated with health professional’s age between 25–34 year (AOR = 0.25, 95% CI = 0.08–0.74) and working in pediatric ward (AOR = 0.39, 95% CI = 0.17–0.90) and in emergency ward (AOR = 0.25, 95% CI = 0.09–0.67) [23]. In Nigeria and Uganda, 75.0% of the staff viewed adverse events as mistakes made by healthcare personnel during patient treatment or management [19].

### Challenges associated with the implementation of patient safety culture

In a qualitative study of health care professionals in two facilities in east Africa, multiple factors influence the ability to implement patient safety measures in the health care facility [28]. It was identified that the proximal cause of patient safety issues is the non-availability of the surgeon to perform an emergency operation, while the distal factors related to the total lack of professionals nationally for distribution to the various health facilities [27]. The distal causes also include material deprivation, lack of teamwork, and poor accountability of management [27]. Health care providers in the Democratic Republic of Congo were particularly challenged in implementing patient safety measures due to arm conflicts and blame games between the various cadres of health care professionals, which resulted in dire consequences [28]. The increased corruption within health organizations and population impoverishment and substance abuse among health staff adversely altered safe care [28].

The out-of-pocket payment strategy used when those patients had to pay for health services directly at the point of the acquisition was an essential factor that adversely affected patient safety practices in the hospital in the Democratic Republic of Congo [28]. In the study using Nigeria as a case study after the outbreak of the corona pandemic, the managerial role was assessed not to positively influence workers’ perspective on patient safety in health care institutions [22], as shown in Table 2. In Ethiopia, community pharmacists also identified that there is an enormous problem related to mistake communication (44.8%) and work pressure (45.0%) [26]. Community pharmacists in Ethiopia showed that 61.5% of the study subjects stated that there was poor communication on the status of inappropriate prescriptions across shifts [26].

**Table 2:** Distribution of factors affecting patient safety and key findings.

Ref	Factors and dimensions	Key finding	Knowledge, awareness, and perception
Aveling et al., 2015 [27]	Shortage of skilled nursing staff, shortage of material resources, lack of access to necessary, limited specialist training, gaps in human resources, higher staff turnover	• The factors associated with patient safety are non-functional equipment, lack of trained maintenance staff, systemic failures, poor budgetary allocations, lack of access to necessary drugs, patient poverty, delays, and other procurement processes.	• Hospital staff offered broadly encompassing and aspirational definitions of patient safety. • Participants identified obstacles across three major themes: material context, staffing issues, and inter-professional working relationships.
Nwosu et al., 2019 [24]	Infection (64.0%), blood transfusion services (34.0%)	• Perceived hospital-acquired infection and abuse of transfusion were important issues for patient safety. • Awareness of institutional policies to prevent surgery at the wrong site (38.8%), only 11.3% practiced policies to reduce the risk of surgery at the wrong site.	• Consultants/specialists were about four times, and resident doctors were about three times more likely to have a good perception of patient safety issues than interns/house officers.
Labat & Sharma, 2016 [28]	The attributes of patient safety culture: paternalistic, blame culture, inefficient support services, low salaries and arm conflicts, corruption, patient poverty, and substance use by staff	• The factors that influence patient safety were human resources and hospital management, healthcare access, paternalistic organizational structure, blame culture, perceived inefficient support services and low salaries, armed conflicts, system failures, a threat to patients and health care workers, increased corruption, population impoverishment and substance abuse among health staff. • Positive outcomes were associated with health workers’ resilience and resourcefulness to address barriers.	• Anesthesia was perceived as the significant issue associated with patient safety and complications. • Perceived OT preparation, hygiene, and collaboration within a multidisciplinary team are essential to safe surgery. • AEs were mainly perceived as HWs responsibility, managed by blame and punishment.
Swart et al., 2015 [25]	Losing patient information, staff mistakes, verbal abuse, hospital-acquired infections, physical abuse, and patient incidents	• Enrolled nurses indicated that current efforts to prevent errors are adequate, and registered nurses obtained high scores in reporting incidents related to patient safety. • Nurses mostly reported medication errors, pressure ulcers, and falls with injury.	• Enrolled nurses (51.0%) rated patient safety as very good, and registered nurses (51.0%) rated it as acceptable. • A significant difference between registered nurses and enrolled nurses’ overall grade of safety (χ2 = 34.1, *p* < 0.001).
Kumbi et al., 2020 [21]	Staff category, work duration, work area, participation in a patient safety program, reporting of adverse events, communication openness, organizational learning, and exchange of feedback about an error	• The highest positive response rate of the items was People supporting one another in this unit (82.2%), while the lowest positive response rate of the item was ‘We have enough staff to handle the workload (27.2%). • Physician category of staff position, hours worked per week, primary work area (surgery and pharmacy), participation in the patient safety program, and adverse event reported showed an association.	• The overall level of patient safety culture was 44.0% (95% CI: 43.3–44.6) and was rated as poor (12.4%), excellent (29.3%), and the positive response rate for each of the items ranged from 22.0% to 85.0%.
Moda et al., 2021 [22]	Management safety priority commitment, competence management, safety empowerment, management safety justice, worker safety commitment, worker safety priority, and risk	• Health worker safety commitment within the healthcare facilities was significantly higher than management safety priority, commitment, and competence. • A significant effect of the management role was found regarding management safety priority, commitment, and competence.	• There is the active promotion of a positive safety climate in healthcare sectors. • Employees are more likely to engage in positive safety behavior.
Gqaleni & Bhengu, 2020 [20]	Hospital-related incidents, patient, care-related incidents, medication-related incidents, blood product-related incidents, procedure-related incidents	• High rates of PSIs, with increased length of stay were observed in multidisciplinary CCUs (49.0%), neonatal CCUs (29.0%) and cardiac CCUs (20.0%), and pediatric CCUs (1.7%). • Blood-related incidents (5.0%) and medication-related events (7.0%) were more minor or insignificant.	• The rating of patient safety incidents was insignificant (18.0%), minor (35.0%), moderate (25.0%), major (12.0%), and catastrophic (10.0%).
Cheikh et al., 2016 [31]	Hospital management support, teamwork, supervisor expectations, and actions, promoting safety, frequency of event reporting, organizational learning, and continuous improvement	• The dimensions that received a lower positive response rate from doctors were “hospital management support for patient safety” (13.9%) and “teamwork within units” (45.4%), while those with the highest positive response included “supervisor expectations and actions promoting safety” (82.3%), and “frequency of event reporting” (84.0%). • For paramedical staff, the dimensions with the highest positive for paramedical staff were “organizational learning and continuous improvement” (67.8%), and the lowest positive response was related to “hospital management support for patient safety” (40.9%).	• Dimension having the most developed score was the perception of “frequency and reporting adverse events” (68.8%), and the lowest score was “management support for safety care” (32.7%). • Overall scores of different dimensions variables between 32.7% and 68.8%. • All of them claimed that the main source of information on the SC was experience (50.5%), medical school (37.7%), general culture (25.8%), and media (22.0%).
Alhassan et al., 2015 [29]	Leadership and accountability, capable workforce, safe environment for staff and patients, clinical care of patients’ improvement of quality and safety	• In health care facilities that received the intervention, staff efforts in increasing patient safety and reducing the risk associated with patient care were significant. • The specific areas that received improvement were leadership accountability and staff competencies.	• Patient safety culture interventions significantly enhanced leadership processes and accountability.
Akologo et al., 2019 [16]	Teamwork, supervisor expectations, and actions, organizational learning, continuous improvement, management support, feedback and communication about the error, openness, staffing, frequency of events reported, non-punitive response to error, the overall perception of patient safety	• Teamwork and organizational learning of the 12 patient safety dimensions had higher scores. • Dimensions with high positive response rates were teamwork (81.5%), organizational learning (73.1%), and low positive response rates (50.0%) were staffing (34.5%), non-punitive response to error (33.9%), and frequency of events reported (45.7%).	• The patient’s safety was rated as excellent (7.0%), very good (43.8%), acceptable (35.0%), poor (13.8%), and failing (1.0%). • In general, perception of the patient safety dimension positively correlated with patient safety culture dimensions for all categories except for staffing.
Yismaw et al., 2020 [26]	Teamwork, physical space, and environment	• A positive response rate was demonstrated in the domains of teamwork (90.2%), physical space and environment (83.1%), mistake communication (44.8%), and work pressure (45.0%). • The overall rating of the pharmacy on patient safety was excellent (33.0%), very good (30.8%), good (25.1%), fair (7.5%), and poor (3.3%).	• Most participants did not carry out any documentation of mistakes. • There is no documentation in 59.0% of cases when a mistake that could have harmed the patient is corrected.
Mohammed et al., 2021 [23]	Type of profession, level of education, work experience, age, hospital type, and working units	• Good patient safety culture was positively associated with working in primary hospitals. • Good patient safety culture was negatively associated with health ages 25–34 years and working in the pediatric and emergency wards. • Health care professionals working in pediatrics (61.0%, AOR = 0.39) and emergency wards (75.0%, AOR = 0.25) are less likely to have a good patient safety culture compared.	• Perceived good patient safety culture (44.8%), teamwork in hospital units (74.1%), and departments (53.1%), and the supervisor’s expectation (51.9%) were positively contributing dimensions to the overall patient safety culture.
Atakora et al., 2021 [17]	The duration of the training, knowledge on patient safety	• Majority (97.5%) had a moderate level of knowledge on patient safety. • There was no significant association between the levels of study and knowledge of clinical year physiotherapy students on patient safety.	• Most respondents (97.5%) had moderate knowledge of patient safety. • High level of knowledge on safety in the workplace (72.5%) and safety of the healthcare system (60.0%)
Mayeng & Wolvaardt, 2015 [14]	Organizational learning, communication, personnel management, staff education, and training, teamwork	• The nurses scored only positive organizational learning following a patient safety incident (62.9%), while doctors scored staff education and training least poorly (58.3%). • Overall patient safety was rated as acceptable (42.4%), very good (28.5%), excellent (14.6%), poor (11.8%), and failing (2.8%). • The nurses’ positive perceptions were significant for perceptions of the causes of patient safety incidents (*p* < 0.003); investigating patient safety incidents (*p* < 0.001); and organizational learning following a patient safety incident (*p* < 0.001).	• There was also a positive perception of nurses and the causes of patient safety incidents, investigation of patient safety incidents, and organizational learning following a patient safety incident. • The community service professionals had a significantly negative perception of the permanent staff on the dimensions: overall commitment to quality dimension; organizational learning following a patient safety incident; and communication about safety issues.
Ente et al., 2010 (19]	Staff knowledge, understanding of medical error, the impact of medical error, availability of remedy service	• Frontline health care professionals knew well about patient safety culture and medical errors. • Staff was aware that errors could cause suffering to the patient and could even lead to death or damage hospitals’ reputations, costing them their job.	• The staff viewed adverse events as mistakes (75.0%) made by healthcare personnel during patient treatment or management.
Gizaw et al., 2018 [18]	Teamwork, supervisors’ expectations, and action, communication openness, feedback and communication about the error, frequency of event reporting, non-punitive response to error, staffing, hospital management support, hospitals handoffs and transfer of the patient and organizational learning, continuous improvement	• Teamwork within the unit is the only area with a higher positive response (79.4%). • The composite percentage of positive responses was the frequency of event reports (28.3%), hospital management support for patient safety (34.7%), hospitals handoffs and transition (41.3%), non-punitive response to error (44.7%), teamwork across the unit (47.4%) and communication openness (48.7%). • Patient safety was significantly associated with non-punitive response to error, teamwork, staffing, unit collaboration, and openness in communication.	• The overall perception of patient safety was 36.8%. • Perception of patient safety practices and teamwork increased across the units.

AE, adverse event; CCU, coronary care unit; HW, health worker; OT, operating theater; PS, patient safety; PSIs, Patient safety incidents

### Factors that promoted patient safety practices in the hospital

Physician profession, hours worked per week, participation in the patient safety program, an adverse event reported, teamwork within the hospital, organizational learning, communication openness, frequency of event reporting, feedback & communication, management support for patient safety, teamwork across hospital and handoffs and transitions were found to be significantly associated with the patient safety culture [21] as shown in Table 2. In the Democratic Republic of Congo, patients identified the need to be insulated from the arm conflicts that ravaged that country as an essential contribution to patient safety and measures to reduce the out-of-pocket payment currently practiced in health care institutions [28]. Increasing the level of nurse education from enrolled to registered nurses showed a positive statistical significance for patient safety as it relates to preventing errors in the unit, losing patient information between nursing shifts, medication errors, ulcers, falls, and patient injury [25]. The staff expressed their willingness to learn more about patient safety and how to prevent medical errors but listed some of the learning methods they would prefer. These include seminars, conferences, symposia, Continuing Medical Education (CME), interactive sessions, short courses, workshops, training aids, and videos using information sources such as the Internet, publications, handouts, and newsletters [19]. In Ethiopia, as a non-punitive response to error increases by one unit (*p*<0.001), the perception of patient safety practice increases by 0.190 [18].

## Discussions

This study synthesized and integrated the status and factors influencing patient safety in healthcare institutions in Africa. Patient safety issues are essential for improving health outcomes, reducing risk, and minimizing the dangers associated with patient care. Patient safety culture, since its inception, has received some concept analysis. It can be described as preventing medical errors, avoidable adverse events, protecting patients from harm or injury, and ensuring a collaborative effort for individual health care providers and integrated solid health care teams [12, 32–34]. These factors related to patient safety in lower-middle-income countries may be individual or professional gaps or negligence, systemic factors or the lack of appropriate knowledge, obsolete equipment, technological failure or misapplication, or the total lack of the requisite resources. Patient safety as a product of health can be attained by ensuring having a positive reporting culture, minimizing error, creating awareness, providing education, ensuring the use of appropriate health care professionals and equipment, adopting a non-penalizing culture, and promoting teamwork [4, 9, 32, 35]. Essentially, the concept of patient safety is to ensure a safe environment for the care of patients and health care professionals and ensure that the risk of injury is minimum [33]. Patient safety practices should be regarded as a culture and become part of healthcare institutions’ everyday service delivery practices [32]. The world health organization insists that the discipline of patient safety ensures coordinated efforts to prevent harm, reduce risk, secure health care processes, and produce a minimal threat to the patients [9, 12, 35].

This study demonstrated the variety of factors that can be attributed to patient safety in health care institutions in Africa. The study further identified the diversity of factors associated with practicing patient safety in health care institutions. These factors are related to communication, error identification, information dissemination, education, teamwork, professionalism, systems, patients, management culture, and leadership. In a systematic review showing interventions studies focused on improving patient safety, five themes were associated with patient safety culture, i.e., error; communication; teamwork and leadership; systems, and situational awareness [36]. The variety of the associated factors demonstrates the comprehensive nature of patient safety, and health care institutions ought to identify these factors as awareness creation and education remain a continuous activity. This indicates that in-service staff training on patient safety attitudes must be a constant process that tackles, evaluates, and promotes each facet of the safety dimension. The categorization of patient safety dimensions must be clearly delineated to promote education and training while allowing for appropriate assessment of the concept using objective tools in health care institutions [36]. Also, recruiting the proper number of skilled staff is essential as staff burnout was identified as an important factor influencing patient safety practices [37].

It was identified that several other factors influence the patient safety culture in health care institutions. These factors range from the individual, system, professional, hospital or institutional, and external factors. The contributions of these factors are varied and multiple. These findings are like those that were reported that some primary factors that seem to affect this culture are well-being, burnout, depression, anxiety, poor quality of life, and stress [10]. These factors were noted to be associated with self-reporting error, service process, error communication, human factors related to healthcare providers, and human factors related to patients (lack of attention, stress, anger, and fatigue), the healthcare environment, technical factors, and poor objective measures of errors [10, 13]. It has been noted that there is variation in the perception and utilization of patient safety culture within health care facilities in Africa. Increasing knowledge and encouraging patient safety culture remain cardinal to positive patient outcomes. The wide variation in the practices and knowledge on patients’ safety culture can be attributable to the variation pertaining to systems, socioeconomic, cultural, professional, and perception of health and health care within various African jurisdictions. These contrasting views of perception of patient safety culture within health care facilities were also reported in another systematic review [38]. Synchrony in ideas by all clinical service providers will aid the eventual outcome of patient safety cultural measures. Standardization of procedures and methods across African countries is essential as those all remain a benchmark for promoting positive patient outcomes and minimizing the risk associated with poor care.

The primary studies did not identify the influence of hospital type, workforce, type of services, and patient safety culture in health care institutions. Patient safety practices must be segregated within these parameters to clearly delineate interventions that will be tailored to improve patient safety and promote patient safety within health care institutions. Therefore, future studies should also focus on the influence of hospital type, workforce, type of services and patient safety culture in health care institutions

This study highlighted the factors associated with patient safety in African health care institutions. It identified the antecedent, influencing factors, and how to promote positive patient safety cultures in those facilities. The study is not without some challenges, as only articles that were published only in the English language were included. Also, conference papers and other studies in grey literature were not included. This might have limited the scope of perspectives related to patient safety in health service delivery. The study did not discriminate against a particular set of health professionals but included all, which might demonstrate the higher heterogeneity of synthesized perspectives.

## Conclusion

This study identified several factors associated with patient safety in African health care institutions. These individual, team, facility, and systematic factors that negatively influence the patient safety culture must be curtailed to promote better patient outcomes while encouraging positive influencers. Personal knowledge can be improved through education, and training, while systematic barriers to patient safety culture are eliminated through coordinated, systematic approaches incorporating multi-factorial viewpoints. We also identified that to achieve a positive patient safety culture within health care facilities, health care managers ought to be conscious of this need and institute measures to promote best practices. Non-punitive action by authorities, investigation of errors, education, communication, and improved knowledge will be helpful. Incorporating patient safety actions in health promotion by educating staff will be critical in promoting the culture in health care institutions. Also, using intervention research techniques to promote best practices crucial to service delivery in these poor resource settings will be critical in promoting patients’ safety culture. Intervention research may promote patient safety culture, error reporting, and awareness of the concept, especially among healthcare providers.

## Supporting information

S1 Checklist PRISMA checklist. (DOCX)

## List of abbreviations

CCU: Coronary Care Unit
CINAHL: Cumulative Index for Nursing and Allied Health Literature
CME: Continue Medical Education
MMAT: Mixed Methods Appraisal Tool
PICO: Patient, Intervention, Comparison, and Outcome
PSI: Patient Safety Incidence
VAP: Ventilator-Associated Pneumonia
WHO: World Health Organization
